# A Pronectin™ AXL-targeted first-in-class bispecific T cell engager (pAXLxCD3ε) for ovarian cancer

**DOI:** 10.1186/s12967-023-04101-x

**Published:** 2023-05-04

**Authors:** Caterina Riillo, Nicoletta Polerà, Maria Teresa Di Martino, Giada Juli, Craig A. Hokanson, Tatjana Odineca, Stefania Signorelli, Katia Grillone, Serena Ascrizzi, Antonia Mancuso, Nicoletta Staropoli, Basilio Caparello, Maria Cerra, Giuseppe Nisticò, Pierosandro Tagliaferri, Roberto Crea, Daniele Caracciolo, Pierfrancesco Tassone

**Affiliations:** 1 Department of Experimental and Clinical Medicine, Magna Græcia University, Catanzaro, Italy; 2 Protelica, Inc, Hayward, CA USA; 3 Giovanni Paolo II General Hospital, Lamezia Terme, Italy; 4 Renato Dulbecco Institute, Lamezia Terme, Italy; 5 S.H.R.O., College of Science and Technology, Temple University, Philadelphia, PA USA

**Keywords:** Pronectins, Bispecific T cell engager, BTCE, BiTe, Ovarian cancer, AXL, Immunotherapy, CD3, Cancer

## Abstract

**Background:**

Pronectins™ are a new class of fibronectin-3-domain 14th-derived (14Fn3) antibody mimics that can be engineered as bispecific T cell engager (BTCE) to redirect immune effector cells against cancer. We describe here the in vitro and in vivo activity of a Pronectin™ AXL-targeted first-in-class bispecific T cell engager (pAXLxCD3ε) against Epithelial Ovarian Cancer (EOC).

**Methods:**

pAXLxCD3ε T-cell mediated cytotoxicity was evaluated by flow cytometry and bioluminescence. pAXLxCD3ε mediated T-cell infiltration, activation and proliferation were assessed by immunofluorescence microscopy and by flow cytometry. Activity of pAXLxCD3ε was also investigated in combination with poly-ADP ribose polymerase inhibitors (PARPi). In vivo antitumor activity of pAXLxCD3ε was evaluated in immunocompromised (NSG) mice bearing intraperitoneal or subcutaneous EOC xenografts and immunologically reconstituted with human peripheral blood mononuclear cells (PBMC).

**Results:**

pAXLxCD3ε induced dose-dependent cytotoxicity by activation of T lymphocytes against EOC cells, regardless of their histologic origin. The addition of PARPi to cell cultures enhanced pAXLxCD3ε cytotoxicity. Importantly, in vivo, pAXLxCD3ε was highly effective against EOC xenografts in two different NSG mouse models, by inhibiting the growth of tumor cells in ascites and subcutaneous xenografts. This effect translated into a significantly prolonged survival of treated animals.

**Conclusion:**

pAXLxCD3ε is an active therapeutics against EOC cells providing a rational for its development as a novel agent in this still incurable disease. The preclinical validation of a first-in-class agent opens the way to the development of a new 14Fn3-based scaffold platform for the generation of innovative immune therapeutics against cancer.

**Supplementary Information:**

The online version contains supplementary material available at 10.1186/s12967-023-04101-x.

## Background

Pronectins are a novel class of binding proteins structurally based on the non-immunoglobulin fourteenth fibronectin type-3 scaffold (14Fn3) [1]. The human fibronectin-3 is an extracellular protein abundant in normal serum [2]. Several examples of functional single domain protein binders tailored upon the 10th domain of FN3, called collectively monobodies, have been reported in the literature since the pivotal work [3, 4]. The 14Fn3 human scaffold, that was selected by bioinformatic analysis [5], can be engineered to confer antibody mimics properties leading to synthetic proteins. Pronectins are similar in binding to monobodies or other single domain antibody mimics [4]. FN3 scaffold structures can be empowered with enhanced binding potency, high specificity and stability by both loops engineered diversity and/or scaffold optimization [6–8]. In addition to their small size (approx. 1/15th of a mAb) that facilitates tumor mass penetration, Pronectins lack disulfide bridges which enhance stability in tumor microenvironment (TME) and high-yield production in *E. coli,* yeast, and microalgae. Moreover, careful selection of their three CDR loops to include only human amino acids may prevent or eliminate immunogenicity. The intrinsic pharmacological properties of Pronectins can also provide meaningful structural diversity and related scale-up to a targeting repertoire from a few thousand to 25 billion new epitope structures. Finally, Pronectins can be engineered as multimers, fusion proteins, and bispecific constructs, therefore opening a wide landscape of potential applications as innovative immune therapeutics for targeting human cancer [9]. While novel approaches, such as Bispecific T cell Engager (BTCE), Chimeric Antigen Receptor (CAR-T), Antibody Drug Conjugated (ADC) targeted monoclonal Antibodies (mAbs) are progressively improving the outcome and survival of patients carrying different hematological malignancies, the successful application of these innovative treatments for solid cancers is still hampered by the lack of specific tumor associated antigen (TAAs), impaired maintenance of T-cell activation at the tumor site and, most importantly, poor penetration within an hostile TME. In this context, Pronectin-based BTCEs (pBTCEs) can overcome some of the above limitations by enhanced stability within the acidic TME and the reduced size while simultaneously targeting and generating a bridge between a TAA expressed on cancer cells and typically the epsilon (ε) subunit of CD3 expressed by T-lymphocytes. This simultaneous engagement of the two antigens leads to immunological synapses, resulting in T-cell activation and MHC-I independent CD3-mediated killing [10].

So far, BTCEs have been mostly engineered using original mAbs scaffolds or fragments. This approach has led to the development of new drugs [11–15], and some of them are already approved for clinical use [10, 16], but the scale up to clinical translation of the selected candidates has been very slow, time-consuming, and highly expensive. Alternative strategies overcoming these limitations would significantly speed-up the selection of promising candidates [17],their production according to Good Manufacturing Practice (GMP) and the early clinical testing, quickly expanding the therapeutic scenario for orphan diseases. A promising alternative option for the generation of BTCEs would include the use of simple, disulfide free, non-immunoglobulin scaffolds with huge and expanding diversity on TAAs binding capacity, and very small sizes that allow deep penetration within a tumor mass. In this light, the Pronectin platform can lead to the construction of tissue agnostic Pronectin-based paratope libraries to be screened on specific TAAs or high throughput cancer tissues microarray, allowing quick and effective selection of immune-targeting constructs for further development. With this aim, we here provide proof of concept findings to validate the use of pBTCEs in the experimental treatment of a humanized in vivo pre-clinical model of Epithelial Ovarian Cancer (EOC), a disease where immunotherapy is still in its infancy [18].

EOC is the most common cause of gynecological cancer death, partially due to the common advanced stage at the diagnosis [19]. Unfortunately, despite these recent advances, the prognosis of EOC patients remains poor, with a 5-years survival of only 29%. EOC patients experience relapse/progression and/or develop platinum-resistant disease with an extremely adverse prognosis. This condition represents therefore a critical unmet need urging innovative therapeutics.

AXL is a tyrosine kinase receptor of TAM receptor family [20]. AXL is overexpressed in several malignancies, including EOC, and confers dismal outcome [21]. After binding to GAS6 and PROS1, AXL undergoes homo or heterodimerization and activates downstream signaling pathways, including PI3K/AKT, MAPK, and JAK/STAT. Furthermore, AXL was found overexpressed on macrophages and dendritic cells [22].

Overall, several evidence shows that AXL signaling plays a crucial role in different hallmarks of cancer as abnormal proliferation and survival, resistance to apoptosis, invasion and metastasis, angiogensis and immune suppression [23].

In particular, in ovarian cancer AXL is upregulated in advanced stage and in metastases as compared with normal ovarian tissue [24, 25]. Furthermore, AXL expression is associated with chemoresistance in patient tumors and cell lines [26, 27]. Interestingly, more recent data indicate that AXL expression exhibits variation across monolayer and/or spheroid ovarian cancer cells models. Indeed, in this experimental context, AXL concentrations could differ by 100 times between chemo-sensitive and chemo-resistant cells [27].

Consistently with these findings, AXL genetic or pharmacological inhibition in ovarian cancer led to impaired migration and metastasis and in increased chemo-sensitivity of both patient derived xenograft and ovarian cell lines [24, 26] and could therefore represent a promising therapeutic target in EOC.

Even if some expression has been described on smooth muscle cells and other cells, there is no current evidence of toxicity induced by off-tumor AXL targeting [28]. AXL is, therefore, an attractive tumor target for immune-therapeutic strategies [23].

Based upon these premises, we developed a novel pBTCE AXL-targeted to activate an immune response against EOC cells. Here we report the in vitro and in vivo testing of this pBTCE as a promising immune-therapeutic agent for this poor prognosis disease.

## Methods

### Development of a first-in-class Pronectin™-based anti-AXL (pAXL × CD3)

A highly specific single domain, non-immunoglobulin anti-AXL Pronectin™, has been generated by isolation bioinformatic screening within the human scaffold libraries of the 14th domain of Fibronectin III (14FN3), as recently reported [29]. AXL54 was selected as the best candidate for targeting purposes. On this scaffold, a first-in-class BTCE (AXL54 [Pronectin]-linker-scFV CD3, pAXLxCD3ε), was developed as innovative anti-tumor compound to be investigated in vitro and in vivo.

Binding affinity and kinetic proprieties of p-AXL were determined as previously described [29].

### Cell lines

ES-2, OVCAR-8, SKOV3 and EFO-21 were purchased by DSMZ. RMG-I cell line was purchased by JCRB Cell Bank (Japanese Collection of Research Bioresources Cell Bank).

ES-2, OVCAR-8, SKOV3 and RMG-I were cultured in RPMI 1640 (Gibco^®^, Thermo Fisher Scientific, Waltham, MA, USA), supplemented with 10% fetal bovine serum (Lonza Group Ltd), 100 U/mL penicillin, and 100 μg/mL streptomycin (Gibco®, Thermo Fisher Scientific), and maintained at 37 °C in a 5% CO_2_ atmosphere. EFO-21 cell line was cultured in RPMI 1640 supplemented with 20% fetal bovine serum, + 2 mM L-glutamine + 1 × MEM non-essential amino acids + 1 mM sodium pyruvate.

### ES-2/LUC cells

ES-2 cell line were plated at 2.5 × 10^5^/ml in a 6 well plate. After over-night (O/N) incubation, 60 μl of Lenti-III-PGK-Luc Control Virus (abm, Canada) containing supernatant was added to cell culture in a final volume of 2 ml in the presence of polybrene at the final concentration of 8 μg/ml. Cell transduction was performed via spinoculation for 50 min at 30 °C. 48 h after transduction, cells were subjected to puromycin selection at 0.25 μg/ml. After antibiotic selection, cells were assessed for the expression of luciferase in the presence of luciferin by IVIS.

### 3D spheroid formation

EOC spheroids formation was achieved by using 96-well U-bottom, ultra-low attachment (ULA) microplates. 1000 ES-2 labelled with Cell-Trace Far Red (Thermo fisher) cells were seeded in 100 µL of complete medium in each well. Spheroid formation was daily monitored by EVOS microscope (Invitrogen by Thermo Fisher). Spheroids were then dissociated and stained with FITC-conjugated anti-AXL antibody and analyzed by flow cytometry.

### Peripheral blood mononuclear cells (PBMC) isolation

PBMCs were isolated by Ficoll-Paque Plus (Cytiva Europe GmbH) density gradient centrifugation and then washed twice in culture medium (RPMI-1640 supplemented with 10% FBS) [30]. T cell isolation was performed through immunomagnetic cell sorting using CD4/CD8 microbeads (MACS Miltenyi Biotec). Human monocytes isolation was performed through immunomagnetic cell sorting using CD14 microbeads (MACS Miltenyi, Biotec) according to the manufacturer instructions.

For macrophage differentiation CD14^+^ monocytes (1 × 10^6^/ml) from healthy PBMCs were seeded in RPMI-1640 medium, 100 ng/ml M-CSF (Miltenyi, Biotec) for 7 days in 5% CO2 at 37 °C. On day 8, the medium was replaced with fresh RPMI-1640 and macrophages were plated with EOC cell lines.

### Fluorescence quantification of AXL target

Quantitation of AXL expression on ES-2, SKOV3, OVCAR-8, RMG-I, and EFO-21 cell lines was performed by flow cytometry by using calibrated microspheres (Quantum Simply Cellular, Bangs Laboratories Inc. Fishers) according to the manufacturer’s protocol. The four populations of microspheres were coated with increasing levels of IgG specific for Fc portion of human IgG1. One drop of each microbeads suspension was mixed with saturating quantities of FITC conjugated AXL antibody for 30 min at 4 °C in the dark. The tubes were washed twice in PBS centrifuged at 2500 ×*g* for 5 min, suspended in 500 μL of PBS and analyzed by flow cytometry. Separately, cell lines were incubated with FITC-conjugated anti-AXL antibody for 30 min at 4 °C in the dark. Samples were washed twice in PBS, centrifuged at 400 ×*g* for 5 min, re-suspended in 500 μL of PBS and analyzed by Flow Cytometry. A calibration curve was generated by converting the mean fluorescence intensity from microspheres into Antibody binding capacity (ABC) using a QuickCal^®^ analysis template provided by Bangs Laboratories. The ABC values of cell lines were determined by comparing their fluorescence intensities against the calibration curve.

### Re-directed T cell cytotoxicity assay

Healthy donors derived PBMCs were labelled with Cell-Trace Violet (Thermo Fisher Scientific) viable marker, according to manufacturer instructions. Labeled PBMCs were co-cultured with EOC cell line at different E:T ratio, in the presence of increasing concentrations of pAXLxCD3**ε** or vehicle for 48–72 h at 37 °C and 5% CO_2_, and then stained with 7-AAD (BD Biosciences). Cytotoxicity was detected by flow cytometry (Attune NxT Flow cytometer, Thermo Fisher Scientific) as 7-AAD^+^/ Cell Trace Violet^−^ cells (%). In the cytotoxicity experiment with T cell depletion, immunomagnetic cell sorting using CD4, CD8 microbeads (MACS Miltenyi Biotec) was performed. In the cytotoxicity experiment with Fc blocking, Fc receptor binding inhibitor antibody (Invitrogen) was added to cell co-culture according to manufacturer instructions.

For 3D re-directed T cell cytotoxicity assay, after EOC spheroids formation 1 × 10^6^ Cell Trace Violet PBMCs were added to each well. Cells were treated with increasing concentration of pAXLxCD3ε or vehicle. After 72 h, spheroids were monitored by microscope scoring. After treatment spheroids were pooled and dissociated with trypsin–EDTA (0.05%). Cells were labelled with 7AAD viable marker for 15 min at room temperature. Finally, cells were analyzed by flow cytometer.

### Bioluminescence T cell re-directed cytotoxicity assay

To perform bioluminescence-based cytotoxicity assay, 1000 ES-2 Luc cells were plated in a 96 well plate with flat bottom. After O/N cell incubation, PBMCs were added to cell culture at 10:1 E:T ratio and treated with increasing concentration of pAXLxCD3ε or vehicle for 72 h. After 3 days, D-luciferin potassium salt (PerkinElmer) was added to cell culture and bioluminescence was detected via luminometer GloMax.

### Direct cytotoxicity assay

EOC cells were plated in the absence of effector cells in a 96 well plate, treated with increasing concentrations of pAXLxCD3ε or vehicle and incubated at 37 °C and 5% CO_2_. After 72 h, 10ul of CCK8 solution (Dojindo Molecular Technologies, Inc.) was added to cell culture, cells were incubated for 4 h and the absorbance at 450 nm was measured using a microplate reader.

### Cell viability assay

EOC cell lines were plated in the absence of effector cells in a 96 well plate, treated with increasing concentration of pAXLxCD3ε or vehicle and incubated at 37 °C and 5% CO_2._ After 72 h cells were detached and cell viability was analyzed by Cell Titer-Glo assay (Promega) according to the manufacturer instructions.

### T cell activation and proliferation

EOC cells were plated for cytotoxicity assays and were incubated with PBMCs at increasing E:T ratio for 24–48 h at 37 °C and 5% CO2. T cells were stained with anti-human CD4 PE-Cy7, CD8 APC-Cy7, CD25 APC, CD69 PE/APC (BioLegend/BD Biosciences), CD107a APC (BD Biosciences, 4 h of incubation). T cells were selected as Far-Red negative, gated for CD4^−^ or CD8^−^ positive, and for CD69, CD25, or CD107a-positive cells. For IFN-γ, TNF-α and perforin intracellular staining, T-ALL target cells and PBMCs were plated at 10:1 E:T ratio in the presence of increasing concentration (0.1 μg/ml, 1 μg/ml, and 2.5 μg/ml) of pAXLxCD3**ε** or negative control. Brefeldin A 10 mg/ml was added, and cells were incubated at 37 °C, 5% C0_2_ for 4 h.

Target and effector cells were washed twice with room temperature 1 × PBS and subsequently stained for 20 min with CD4 PE-Cy7 and CD8 APC-H7 (BD Biosciences).Cells were washed twice with 1 × PBS, fixed with reagent A (Nordic- MUbio) for 15 min protected from light, then washed with 1 × PBS, permeabilized with Reagent B (Nordic- MUbio) and stained with anti- TNF-α PE (BD Biosciences), anti-IFN-γ BV-421 (BD Biosciences), anti-perforin PECF594 (BD Biosciences), and anti-IL-2 PE-Cy7 mAbs (Thermo Fisher Scientific) for 15 min at RT protected from light. After incubation, samples were washed in 1 × PBS and analyzed by ATTUNE NxT flow cytometer (Thermo Fisher Scientific).

T Cell proliferation was measured using Cell Trace Violet (Thermo Fisher Scientific) staining and Cell Trace Violet dilution analysis. Briefly, Cell Trace Violet stained PBMCs were co-cultured with EOC cells as previously (where?REF) described for cytotoxicity assay. After 5 days of co-culture, cells were washed with PBS and acquired immediately by flow cytometry (ATTUNE NxT, Thermo Fisher Scientific).

### pAXLxCD3ε *plus* Poly-ADP-ribose polymerase inhibitors (PARPi) combinatorial approach

ES-2 cells were plated in a 48 well plate alone and in co-culture with Cell-Trace Violet PBMCs in a 10:1 E:T ratio at 37 °C with 5% of CO2. ES-2 alone and the co-cultured with PBMCs were treated with Olaparib (1 μM), pAXLxCD3ε 1 μg/ml and the combo (Olaparib + pAXLxCD3ε). After 72 h, cells were detached and stained with 7AAD. Cells were analyzed by flow cytometry.

### In vivo studies

In vivo experiments were performed according to standard guidelines and approved protocols by the National and Institutional Animal Committee. Six-to-eight-week-old female NSG (NOD.Cg-PrkdcscidIl2rgtm1Wjl/SzJ) mice were purchased from Charles River Laboratories (Wilmington, MA, USA). Animals were euthanized if signs of disease-related symptoms or graft-versus-host disease (GvHD) were developed.

In order to set disseminated EOC in vivo model, ES-2 cells were tripsynized and washed twice with medium. Then, 10 × 10^6^ ES-2 cells were resuspended in 200ul of PBS 1X and intraperitoneally (IP) injected. At day 14, 15 × 10^6^ PBMCs were IP injected into each mouse. Three days after PBMCs engraftment, mice were randomized into 2 groups of treatment: (1) vehicle (2) 0.1 mg/kg pAXLxCD3ε and mice were daily IP injected. Mice were monitored for tumor burden and ascites. At the day of sacrifice, peritoneal effusion from each mouse was collected, lysed, and stained with anti-human CD45-B510 and anti-human AXL-FITC. The peritoneal effusion was analyzed by flow cytometry and the presence and the percentage of EOC cells was analyzed.

In parallel, a subcutaneous (SC) xenografted in vivo model was obtained by inoculation of mice with 10 × 10^6^ ES-2 cells in the dorsal left flank of each animal. At day 14, 15 × 10^6^ PBMCs were IP injected, and the treatment started when palpable tumors were observed (100–200 mm^3^). Tumor sizes were measured by a caliper. The tumor volume was calculated using the formula [tumor volume = (W (2) × L)/2]. Mice were sacrificed when tumor reached > 2000 mm^3^ as previously described [31, 32]. At the sacrifice, blood samples were collected. Red blood cells lysis was performed, and cells were stained with anti-human CD45 BV510 (BD) and anti-human CD3 PerCp-Cy5 (BD) to evaluated PBMCs engraftment.

### Histology and immunohistochemistry

Retrieved tumors from animals were fixed in 4% buffered formaldehyde then, after least 24 h, washed, dehydrated, and embedded in paraffin. For immunohistochemistry, 3-µm-thick tumor slices were deparaffinized and treated with Epitope Retrieval Solution 2 (EDTA-buffer pH 8.8) at 98 °C for 20 min. After washing, peroxidase was blocked by exposing samples to the Bond Polymer for 10 min. All procedures were performed with the Autostainer 48 Automated Immunohistochemistry instrument (Agilent Technologies). Tissues were washed again and then incubated with the monoclonal mouse anti-human CD3 (Dako, clone F7.2.38). Tissues were then incubated with DAB-Chromogen (8 min) and slides were counterstained with haematoxylin (12 min). Light microscopy analysis was performed by an optical microscope AXIO Scope.A1 (Zeiss Oberkochen, Germany).

### Statistical analysis

Each in vitro experiment was performed at least 3 times. Values are expressed as means ± SD/SEM. Statistical evaluation between two groups differences were performed with Student’s *t*-test by GraphPad software (www.graphpad.com). Graphs were obtained using Graphpad Prism version 6.0. Effects were considered statistically significant when p value was less than 0.05.

## Results

### Expression of AXL-receptor on EOC cells

**Fig. 1:**
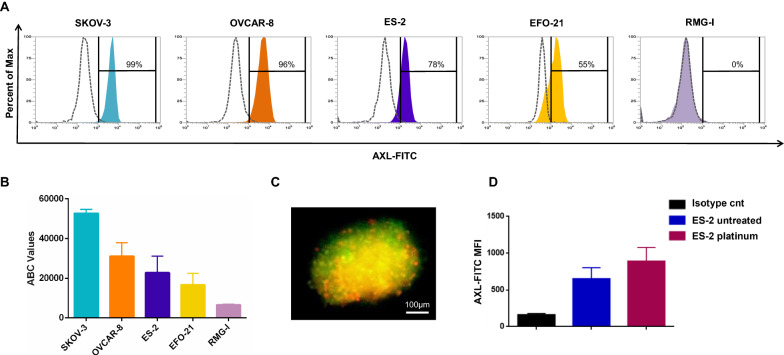
AXL receptor expression. **A** Representative FACS data of AXL binding on EOC cell lines. **B** Graphical representation of Antigen Bounding Count (ABC) evaluated on EOC cell lines by flow cytometry. **C** Representative immunofluorescence microscopy image of AXL (green) expression on the surface of EOC spheroids (red). **D** MFI (median fluorescence intensity) of AXL on ES-2 untreated cells and ES-2 platinum pre-treated cells compared to isotype control

AXL-expressing and not-expressing EOC cells, including serous (SKOV-3, OVCAR-8 and EFO-21) and clear-cell (ES-2 and RMG-I) cell lines, were selected based on the AXL RNA expression level according to the Cancer Cell Line Encyclopedia (CCLE) dataset (https://depmap.org/portal/interactive/) (Additional file 1: Fig. S1A). In the selected cell lines, the expression of AXL receptor was assessed by a fluorochrome-conjugated mAb through flow cytometry. As shown in Fig. 1A, all the EOC cell lines tested, except RMG-I, express AXL at high intensity on the membrane surface. On these cells, the antigen bounding count (ABC) was also investigated by flow cytometry. Interestingly, the median range of ABC was 36.400 and the highest amount of antigen density was found on SKOV-3 (ABC: 53.000) (Fig. 1B). Moreover, AXL expression was detected on 3D cell culture model of EOC through immunofluorescence microscopy analysis (Fig. 1C).

On the basis of the current evidence that AXL tyrosine kinase receptor is strongly involved in the development of chemo-resistance in EOC, AXL expression was evaluated on ES-2 platinum-pre-treated (10 μM) cells by flow cytometry. Interestingly, an higher expression of AXL was found on platinum pre-treated ES-2 cell line as compared to ES-2 untreated cells (Fig. 1D).

Taking together, these data demonstrate that AXL is highly expressed by both EOC cells representing therefore an attractive target for the development of novel immune therapeutics**.**

### pAXLxCD3 ε induces T cell re-directed cytotoxicity on EOC cells

**Fig. 2:**
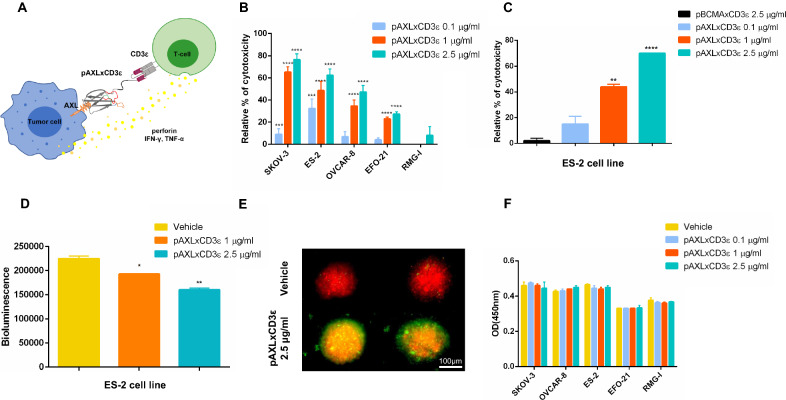
pAXLxCD3ε in vitro T cell re-directed cytotoxic activity. **A** Graphical representation of pAXLxCD3ε structure and mechanism of action. **B** Relative percentage (%) of mediated killing- represented as % of 7AAD negative and AXL positive cells (SKOV3, ES-2,OVCAR-8 and EFO-21) and AXL negative (RMG-I) ovarian cancer cell lines co-cultured with PBMC from healthy donor at E:T ratio 10:1 in the presence of increasing concentrations (0.1 μg/ml, 1 μg/ml and 2.5 μg/ml) of pAXLxCD3ε or vehicle, 72 h after treatment. **C** Relative % of T cell mediated killing on ES-2 cells co-cultured with healthy donor-derived PBMC at E:T ratio 10:1 in the presence of increasing concentrations of pAXLxCD3ε (0.1 μg/ml, 1 μg/ml and 2.5 μg/ml) or negative control (pBCMAxCD3ε 2.5 μg/ml) 72 h after treatment. **D** Viability measured as bioluminescence value of ES-2 LUC cells co-cultured with PBMC from healthy donor at E:T ratio 10:1 in presence of vehicle or increasing concentrations of pAXLxCD3ε (0.1 μg/ml, 1 μg/ml and 2.5 μg/ml) 72 h after treatment. **E** Representative immunofluorescence microscopy image of EOC spheroids (Red) co-cultured with healthy donor PBMC (green) at E:T ratio 10:1 and treated with vehicle or pAXLxCD3ε 2.5 μg/ml. **F** Cell viability measured as absorbance at OD (optical density 450 nm) of EOC cells treated with increasing concentrations of pAXLxCD3ε (0.1 μg/ml, 1 μg/ml and 2.5 μg/ml) in the absence of effector cells. Student’s t-test was applied to calculate statistical significance *p < 0.05, **p < 0.01, ***p < 0.001

pAXLxCD3ε was designed and subsequently purified as previously described [32]. A graphical representation of the molecule and its mechanism of action is reported in Fig. 2A. The effectiveness of pAXLxCD3ε to re-direct T cell mediated cytotoxicity was assessed in vitro by flow-cytometry. PBMC derived from healthy donors were co-cultured with EOC cell lines expressing different level of AXL (ES-2, SKOV3, OVCAR-8 and EFO-21) and with AXL not-expressing cells (RMG-I) at different Effector:Target (E:T) ratio (data not shown) in the presence of increasing concentration of pAXLxCD3ε or vehicle. The E:T of 10:1 was next selected since no significant activity was detected at lower E:T ratio. pAXLxCD3ε induced strong dose- and antigen-dependent killing of AXL-expressing cells. After 72 h of co-culturing, the killing activity of the BTCE at 2.5 μg/ml was about 80% on SKOV-3, 60% on ES-2, 53% on OVCAR-8 and 30% on EFO-21 (Fig. 2B-C). To demonstrate that T cell re-directed cytotoxicity is indeed dependent on binding of AXL (EOC cells) and CD3 (T lymphocytes), B cell maturation antigen (pBCMA)-xCD3ε BTCE was used as negative control on ES-2 cells (BCMA negative, AXL positive). ES-2 cells were co-cultured with PBMCs at 10:1 E:T ratio in the presence of increasing concentration of pAXLxCD3ε and pBCMAxCD3ε. As shown in Fig. 2C no significant T cell mediated killing was detected on ES-2 cell line treated with pBCMAxCD3ε. Moreover, to underline the target specific activity of pAXLxCD3ε, a T cell mediated cytotoxic experiment was performed by co-culturing a multiple myeloma (MM) cell line AMO-1, which does not express AXL but express BCMA, (Additional file 2: Fig. S2A) with healthy donor-derived PBMC at 10:1 E:T ratio and treated with pAXLxCD3ε 2.5 μg/ml or increasing concentration of pBMCAxCD3ε ranging from 0.1 to 2.5 μg/ml for 72 h. As reported in Additional file 2: Fig. S2B, while a dose dependent cytotoxicity was observed on the MM cell line treated with pBMCAxCD3ε, no significant activity was observed on the same cell line exposed to pAXLxCD3ε, 2.5 μg/ml.

T-cell mediated killing was then evaluated on ES-2 stably expressing Luciferase (LUC) reporter gene, via bioluminescence assay. pAXLxCD3ε was able to induce a strong dose dependent cytotoxic effect on ES-2 LUC cells co-cultured with PBMC from healthy donors at 10:1 E:T after 72 h of co-culture (Fig. 2D). Furthermore, to recapitulate the context of coelomic and trans-coelomic EOC disease, [33] a spheroid model of the disease was developed. PBMCs from healthy donors were co-cultured with EOC spheroids for 3 days and treated with increasing concentrations of the BTCE or vehicle. EOC cells were stained with Cell-Trace Far Red and pAXLxCD3ε-dependent T cell infiltration was evaluated through immunofluorescence. Interestingly, pAXLxCD3ε recruited T lymphocytes into the spheroid core and induced a potent anti-tumor activity against EOC cells in vitro (Fig. 2E).

To evaluate if pAXLxCD3ε could induce killing of EOC cells in the absence of effector cells, EOC cells were exposed to increasing concentration of the BTCE or vehicle. Direct cytotoxicity and metabolic activity of EOC cells were evaluated in vitro. As expected, no direct cytotoxicity, nor perturbation of metabolic activity induced by pAXLxCD3ε was found on all EOC cells (Fig. 2F; Additional file 3: Fig. S3A), demonstrating that antitumor activity indeed relies on T-cell engagement.

Moreover, to evaluate if pAXLxCD3ε was able to induce cytotoxicity against healthy donor derived lymphocytes, the surface expression of AXL was evaluated by flow cytometry on the T cells (CD4 and CD8) and an in vitro cytotoxic assay was performed by treating the healthy donor lymphocytes with increasing concentrations of the BTCE. As reported in the Additional file 4: Fig. S4A, AXL expression wasn’t detected on T lymphocytes and accordingly no significant cytotoxicity was observed on the same T lymphocytes (Additional file 4: Fig. S4B).

These results demonstrate that pAXLxCD3ε is capable to engage T-lymphocytes and selectively kills AXL-expressing EOC cells as shown by experiments performed in both bi-dimensional and 3D models of EOC in vitro*.*

### pAXLxCD3ε recruits and activates T cell against EOC cells

**Fig. 3:**
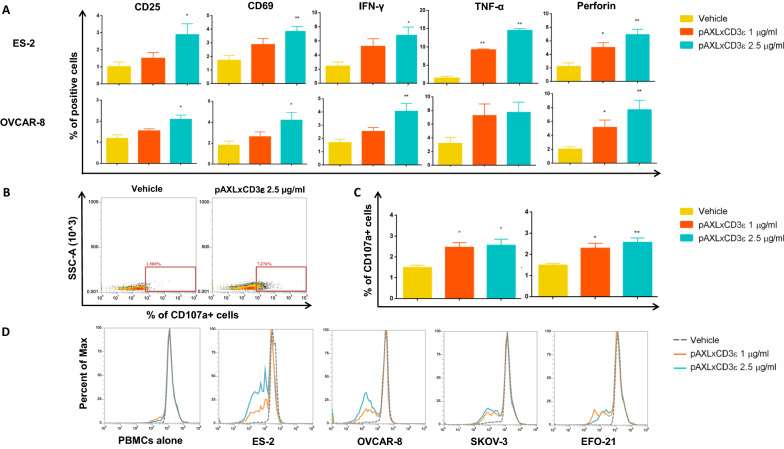
pAXLxCD3ε BTCE in vitro functional activity. **A** Early and late T cell activation markers (CD25 and CD69), cytokine release (IFN-y and TNF-α) and cytotoxic enzyme production (perforin). on CD8 T-lymphocytes co-cultured with AXL positive cells (ES-2 and OVCAR-8) at E:T ratio 10:1 in presence of vehicle or increasing concentrations of pAXLxCD3ε. **B** Representative FACS data of CD107a increase on T-lymphocytes co-cultured with AXL expressing cells (OVCAR-8) treated with pAXLxCD3ε 2.5 μg/ml or vehicle. **C** CD107a dose-dependent increase on T-lymphocytes co-cultured with AXL expressing cells at E:T ratio 10:1 in presence of vehicle or increasing concentrations of pAXLxCD3ε. **D** Proliferation of T cells alone or co-cultured with AXL positive cells at E:T ratio 10:1 in presence of increasing concentrations of vehicle or pAXLxCD3ε. Student’s t-test was applied to calculate statistical significance *p < 0.05, **p < 0.01, ***p < 0.001

We investigated the capability of the BTCE to activate T lymphocytes in the presence of EOC cells. As shown in Fig. 3A, in response to CD3 stimulation and AXL binding, a dose-dependent up-regulation of surface activation markers (CD25 and CD69) was observed on T-cells co-cultured with EOC cells. The production of inflammatory cytokines and other molecules involved in T cell mediated killing, such as IFN-γ, TNF-a, and perforin was next evaluated in T lymphocytes co-cultured with EOC cells at 10:1 E:T ratio in the presence of increasing concentrations of pAXLxCD3ε or vehicle. Importantly, the simultaneous engagement of CD3 and AXL induced a dose-dependent increase of cytokine production (Fig. 3B, C). Finally, proliferation of T cell co-cultured with EOC cells exposed to increasing concentrations of the BTCE was assessed. Consistently with the previously described in vitro data, treatment with pAXLxCD3ε resulted in strong induction of T cell degranulation (Fig. 3B, C) and proliferation (Fig. 3D).

Taken together, these results demonstrate that pAXLxCD3ε induces a dose-dependent specific activation of T lymphocytes against AXL receptor-expressing EOC cells.

### Olaparib enhances pAXLxCD3ε cytotoxicity against EOC cells

During the last decades PARPi have revolutionized the treatment landscape of EOC. The mechanism underlying PARPi efficacy is still under investigation [34, 35] Indeed, beside the synthetic lethality in HR (homologous recombination)-deficient tumor cells, PARPi have been considered to exert their anti-tumor activity also by recruiting CD8 + T lymphocytes via STING pathway enhancing anti-tumor immune response by increasing the production of IFN-y, regardless of BRCAness [34].

BTCEs work by harnessing the patient own immune system to induce T cell mediated cytotoxicity against malignant cells through the production of inflammatory cytokines such as TNF-α and IFN-γ and the release of perforin and granzyme which in turn led to the killing of cancer cells. On this basis, we hypothesized that PARPi may strengthen the T- cell mediated anti-tumor effects induced by pAXLxCD3ε against EOC cells.

**Fig. 4:**
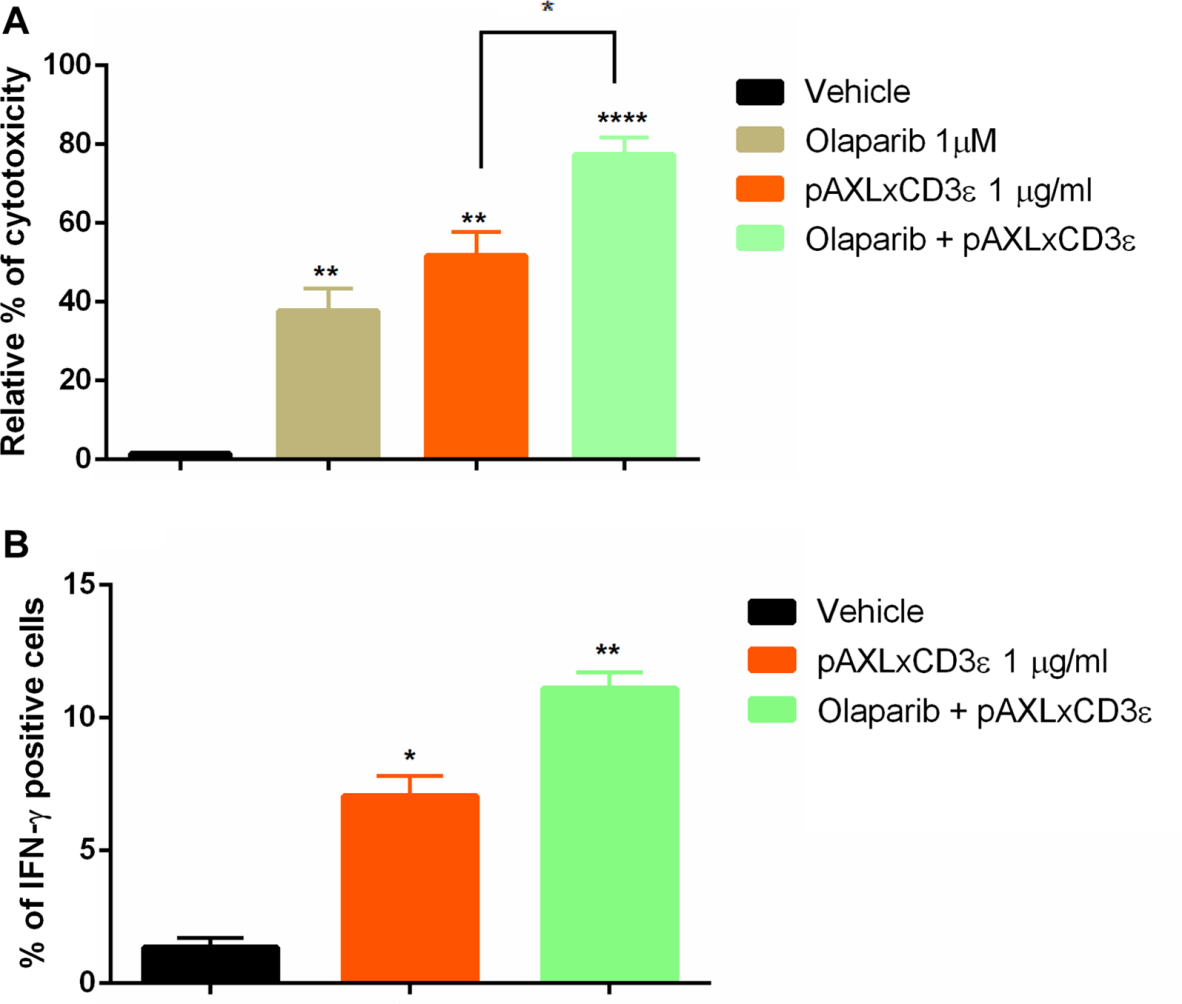
pAXLxCD3ε and Olaparib combinatorial approach. **A** Relative % of T cell mediated cytotoxicity of ES-2 cells co-cultured at 10:1 E:T ratio in the presence of vehicle,1 μg/ml of pAXLxCD3ε and Olaparib 1 μM + pAXLxCD3ε 1 μg/ml. **B** % of IFN-y production in T cells co-cultured with ES-2 cells at 10:1 E:T ratio in the presence of vehicle,1 μg/ml of pAXLxCD3ε and Olaparib 1 μM + pAXLxCD3ε 1 μg/ml. Student’s *t*-test was applied to calculate statistical significance *p < 0.05, **p < 0.01, ***p < 0.001. p values are calculated by comparing PBMC co-cultured with ES-2 cells treated with vehicle to co-cultures of PBMC + target cells treated with pAXLxCD3ε or Olaparib or pAXLxCD3ε+ Olaparib. Relative toxicity is calculated by normalizing pAXLxCD3ε, Olaparib and pAXLxCD3ε plus Olaparib treated cells co-cultured with PBMC on negative control (co-cultures of ES-2 + PBMC exposed to vehicle) placed equally to zero

At this aim, ES-2, a clear cell EOC cell line, wild type (WT) for BRCA 1/2 was co-cultured with PBMCs at 10:1 E:T ratio and then treated with increasing concentrations of Olaparib alone, pAXLxCD3ε alone, or the combination of Olaparib *plus* pAXLxCD3ε, or vehicle. After 72 h, T cell re-directed cytotoxicity was assessed by flow cytometry. The treatment with Olaparib 1 uM *plus* pAXLxCD3ε improved the cytotoxicity on ES-2 cells as compared to pAXL x CD3 alone (Fig. 4A) Moreover, the combinatory treatment with Olaparib 1 uM *plus* pAXLxCD3ε was able to induce a significant increase of the IFN-γ production in T lymphocytes respect than Olaparib and the BTCE alone (Fig. 4B).

These data demonstrate a synergistic effect induced by Olaparib in combination with pAXLxCD3ε suggesting a rationale for the translation of this approach in the context of BRCA WT clinical settings.

### pAXLxCD3ε significantly reduces tumor growth in vivo

**Fig. 5:**
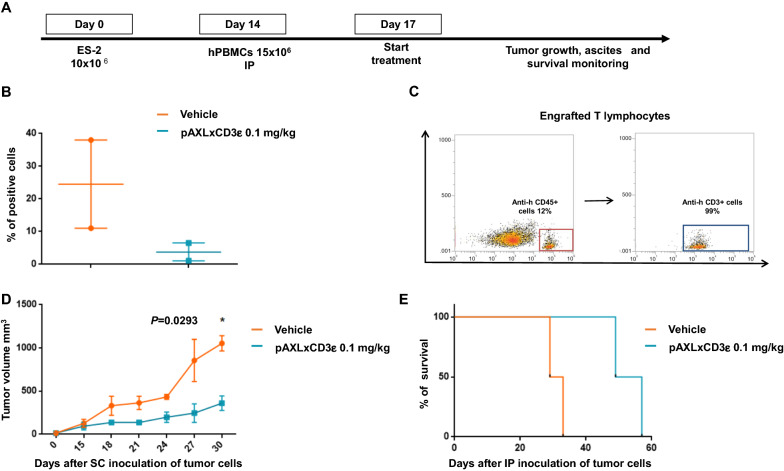
pAXLxCD3ε in vivo activity. **A** Schematic representation of in vivo EOC xenograft model. **B** Percentage (%) of malignant cells in the ascites of NSG mice treated with pAXLxCD3ε 0.1 mg/kg compared to mice treated with vehicle alone. **C** Representative FACS dot plot of T cell engraftment evaluated on the day of sacrifice on (intracardiac) IC blood sample of treated mice. **D** Tumor volume curve of mice treated with pAXLxCD3ε 0.1 mg/kg as compared to vehicle alone **E** Survival curves (Kaplan- Meier) of mice treated with pAXLxCD3ε BTCE 0.1 mg/kg as compared to vehicle alone

As proof-of-concept of anti-tumor in vivo activity of pAXLxCD3ε, different xenograft models of human EOC were settled (Fig. 5A). In a first model recapitulating the human intraperitoneal disease, NSG mice were intraperitoneal (IP) engrafted with ES-2 cells and, 2 weeks later, also injected with human healthy donor derived PBMCs. Three days later, animals were daily treated with the BTCE or control vehicle for 14 consecutive days. Body weight and ascites development were daily monitored. Peritoneal ascites from each animal were collected and analyzed for detection of malignant cells by flow cytometry. Importantly, a significantly higher percentage of EOC cells was found in peritoneal ascites of animals treated with vehicle alone as compared to the group treated with pAXLxCD3ε (Fig. 5B). To evaluate cell engraftment, peripheral blood samples from mice were collected and then stained for CD3-fluorochrome conjugated antibody and finally analyzed by flow cytometry. As shown in Fig. 5C a great human T cell engraftment was obtained.

A second EOC in vivo model was generated by subcutaneous (SC) engraftment of ES-2 cells in the dorsal left flank of mice. 2 weeks later, PBMCs were IP injected and 3 days later were daily IP treated with pAXLxCD3ε or vehicle alone for 14 consecutive days. A significant growth inhibition of SC tumors in mice treated with the BTCE was observed as compared to control group. This anti-tumor effect of the BTCE also translated into a significant prolonged survival of treated animals (Fig. 5D, E).

**Fig. 6:**
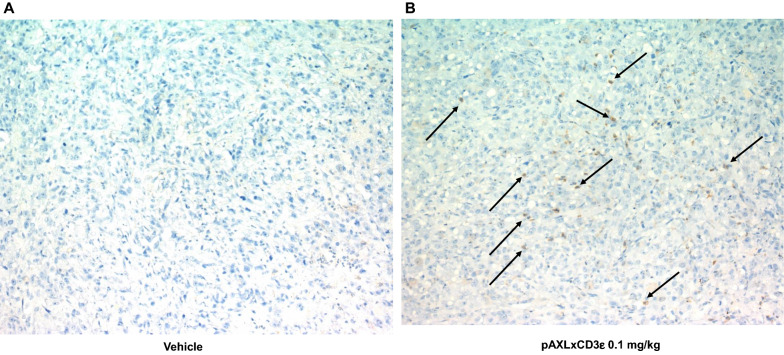
**A** Immunohistochemistry staining of CD3 lymphocytes in explanted tumor from mice treated with vehicle + PBMCs. **B** Immunohistochemistry staining of CD3 lymphocytes in explanted tumor from mice treated with pAXLxCD3ε 0.1 mg/kg. Arrows indicate the stained CD3 lymphocytes

Retrieved tumors from animals were evaluated by immunohistochemistry (IHC) for CD3 T lymphocytes. IHC staining reveled an abundant infiltration of CD3 positive cells in the core of pAXLxCD3ε treated tumors as compared to control (Fig. 6A, B), indicating the effective engagement exerted by our BTCE of human T-lymphocytes at the tumor site further supporting the mechanistic insights from in vitro studies.

Taken together, these in vivo results provide proof-of-concept of anti-tumor activity of pAXLxCD3ε against EOC cells in advanced stage of disease.

## Discussion

BTCEs are a class of novel therapeutics designed to generate an immunological synapsis [36] that activates anti-tumor cytotoxicity in an MHC independent manner, independently from co-stimulatory mechanisms [10]. Since the introduction of Catumaxomab that has been subsequently withdrawn for commercial reasons [37] and Blinatumomab (CD19xCD3ε), approved by FDA in 2014 [38], a variety of BTCEs with different structure were preclinically developed and investigated against hematological and solid malignancies [39, 40]. However, so far, no BTCEs have been approved for the treatment of solid tumors. Indeed, the major hurdles to the BTCEs translation into the treatment of solid tumors are the lack of restricted (specific) TAAs to avoid on-target off-tumor toxicities, and the poor penetration of therapeutics into the tumor mass within a hostile TME. Several TAAs are in fact shared between tumors and healthy tissues, and malignant cells in solid tumors are enveloped in a tumor-prone complex acidic TME that silences immune effectors and polarizes TAMs to a tumor-supporting phenotype, further impairing T cell infiltration [41].

In this context, the tyrosine-kinase receptor AXL may represent a promising target for the development of immune-therapeutics. AXL is in fact differentially expressed among cancer and healthy tissues and its overexpression correlates with worse prognosis in several tumor types, wherein it plays a crucial role in cell growth and migration, in epithelial-mesenchymal transition (EMT), and chemoresistance [23]. Furthermore, emerging findings indicate a key role of AXL in immunosuppressive tumor-microenvironment remodeling and in enhancing immune escape, including MHC-I down-regulation and induction of PD-1/PDL-1 expression [42].

Among healthy cells, AXL expression is detectable in Sertoli, Prostatic glandular, Fibroblast, Kupffer, Smooth muscle and Langerhans cells. A membrane expression of AXL is also documented in plasmacytoid dendritic cells (DC) and natural killer cells (NK) (The Human Protein Atlas). However, the results from a phase 1/2 clinical trial conducted with Enapotamab Vedotin, an anti-AXL antibody coupled with the auristatine E, in non-small cell lung cancer (NSCLC) patients, demonstrated a manageable safety profile, suggesting no critical concerns about AXL off-target effects in cancer patients [43].

On this basis, we developed the first-in-class pAXLxCD3ε which showed an efficient T cell engagement in vitro and in vivo against EOC. This is a heterogeneous disease that includes a variety of histologic types with differential sensitivity/resistance to standard treatments and is characterized by high rates of peritoneal diffusion and metastatic spreading. To date, no specific immunotherapy have been approved for the treatment of EOC, but several immune-based approaches are currently under investigation. The development of effective immune-based strategies to improve the poor therapeutic armamentarium against this aggressive disease can be, therefore, of major clinical relevance. Our pre-clinical data demonstrate that AXL is expressed on the surface of EOC regardless of histologic type and including clear cell tumors that are malignancies highly refractory to medical treatments. In these malignancies, pAXLxCD3ε can, therefore, activate T cell response against tumor. Interestingly, the combination of pAXLxCD3ε with the PARPi Olaparib enhanced IFN-y production by T lymphocytes co-cultured with EOC cells, increasing the anti-tumor activity of the BTCE. This finding is consistent with the recent evidence suggesting that PARPi induce the accumulation of DNA in the cell cytosol, perceived by cGAS that in turn activates the STING pathway [34]. Even if PARPi response and toxicity, as other anti-tumor agents, may undergo inter-individual variability which deserves to be carefully evaluated [44, 45], these events led to IFN-y secretion and promotion of anti-tumor immune response providing a strong rationale for combining DNA damaging based approaches and immunotherapy [46, 47].

A major insight of our work is the use of a first-in-class pAXLxCD3ε characterized by a low molecular weight. Due to its small size, pAXLxCD3ε is prone to a rapid renal clearance which, even if requires continued infusion, lowers the risk of life-threatening adverse events, and allows an easier clinical management of cytokine release syndrome (CRS) and neurotoxicity events. Moreover, the low pAXLxCD3ε molecular weight may facilitate the penetration of the tumor mass ensuring the targeting of cancer cells, the recruitment and activation of T lymphocytes infiltrating the tumor. Finally, its small structure could allow a quick and cost-effective production in *E. coli* and yeast system. Taking together, we believe that data reported here support these theoretical assumptions by demonstrating effective lymphocyte recruiting and powerful activity in different in vivo models.

## Conclusions

In conclusion and to our knowledge, our findings demonstrate the efficacy of a novel non-immunoglobulin FN3 derived-construct targeting AXL in different in vitro and in vivo models of EOC. Our data provide the rational for clinical grade development of pAXLxCD3ε paving the path to a Phase I study in refractory patients. We believe that our results may also open the way for the generation of a platform of new Pronectin-scaffold constructs as innovative immune-therapeutics for the experimental treatment of human cancer.

## Supplementary Information

**Additional file 1: Figure S1.** AXL RNA levels on different EOC cell lines from Cancer Cell Line Encyclopedia (CCLE) dataset.

**Additional file 2: Figure S2.** A) Representative FACS data of AMO-1 cell line stained with anti-human BCMA and anti-human AXL antibody. B) Relative percentage (%) of killing of cells negative for AXL and positive for BCMA (AMO-1) co-cultured with healthy donor-derived PBMCs at E:T ratio 10:1 in the presence of increasing concentrations (0.1 μg/ml, 1 μg/ml and 2.5 μg/ml) of pBMAxCD3ε, pAXLxCD3ε 2.5 μg/ml or vehicle at 72 h after treatment.

**Additional file 3: Figure S3.** A) Percentage (%) of cell viability based on quantification of ATP present in EOC cells treated with increasing concentrations of pAXLxCD3ε (0.1 μg/ml, 1 μg/ml and 2.5 μg/ml) in the absence of effector cells.

**Additional file 4: Figure S4.** A) anti-AXL flow cytometry-based staining on CD4 and CD8 T lymphocytes from 3 different healthy donors. B) Cytotoxicity on healthy donors derived PBMCs treated for 72 hours with increasing concentration of pAXL xCD3 BTCEε (0.1 µg/ml, 1 µg/ml and 2.5 µg/ml).

## Data Availability

Cancer Cell Line Encyclopedia (CCLE) dataset (https://depmap.org/portal/interactive/).
