# Supplementary figures and images for: A Pronectin™ AXL-targeted first-in-class bispecific T cell engager (pAXLxCD3ε) for ovarian cancer
Source: J Transl Med. 2023 May 4;21:301. doi: 10.1186/s12967-023-04101-x (PMC10161629; doi:10.1186/s12967-023-04101-x)

A

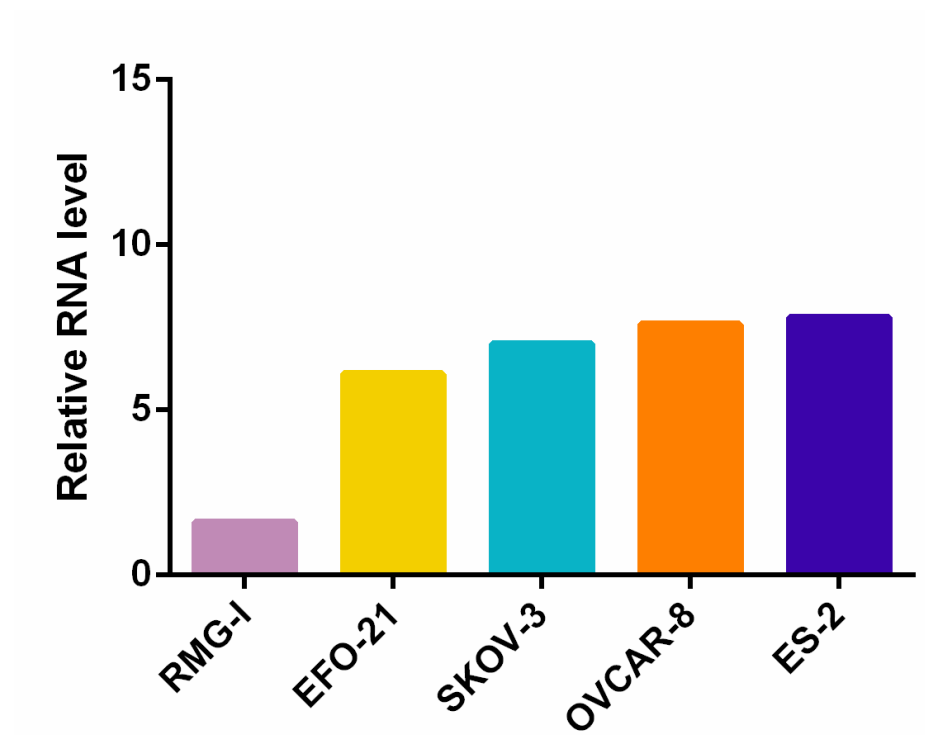

Supplement: Supplementary file 1 — Additional file 1: Figure S1. AXL RNA levels on different EOC cell lines from Cancer Cell Line Encyclopedia (CCLE) dataset. [file 12967_2023_4101_MOESM1_ESM.pdf]

**A**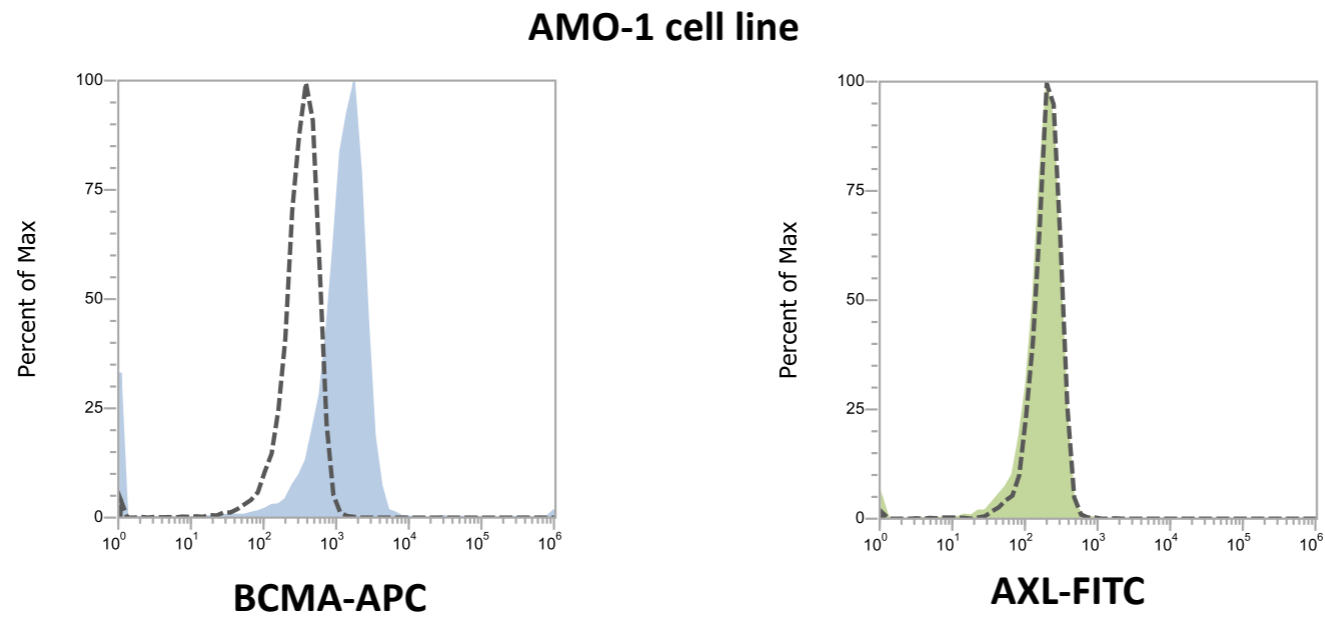**B**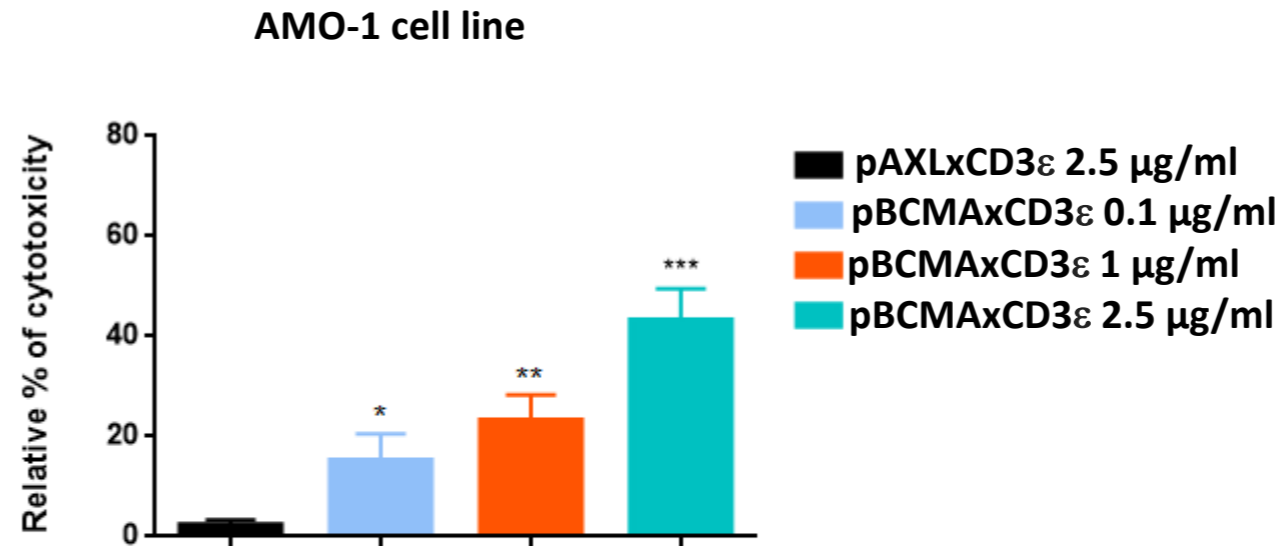

Supplement: Supplementary file 2 — Additional file 2: Figure S2. A) Representative FACS data of AMO-1 cell line stained with anti-human BCMA and anti-human AXL antibody. B) Relative percentage (%) of killing of cells negative for AXL and positive for BCMA (AMO-1) co-cultured with healthy donor-derived PBMCs at E:T ratio 10:1 in the presence of increasing concentrations (0.1 μg/ml, 1 μg/ml and 2.5 μg/ml) of pBMAxCD3ε, pAXLxCD3ε 2.5 μg/ml or vehicle at 72 h after treatment. [file 12967_2023_4101_MOESM2_ESM.pdf]

A

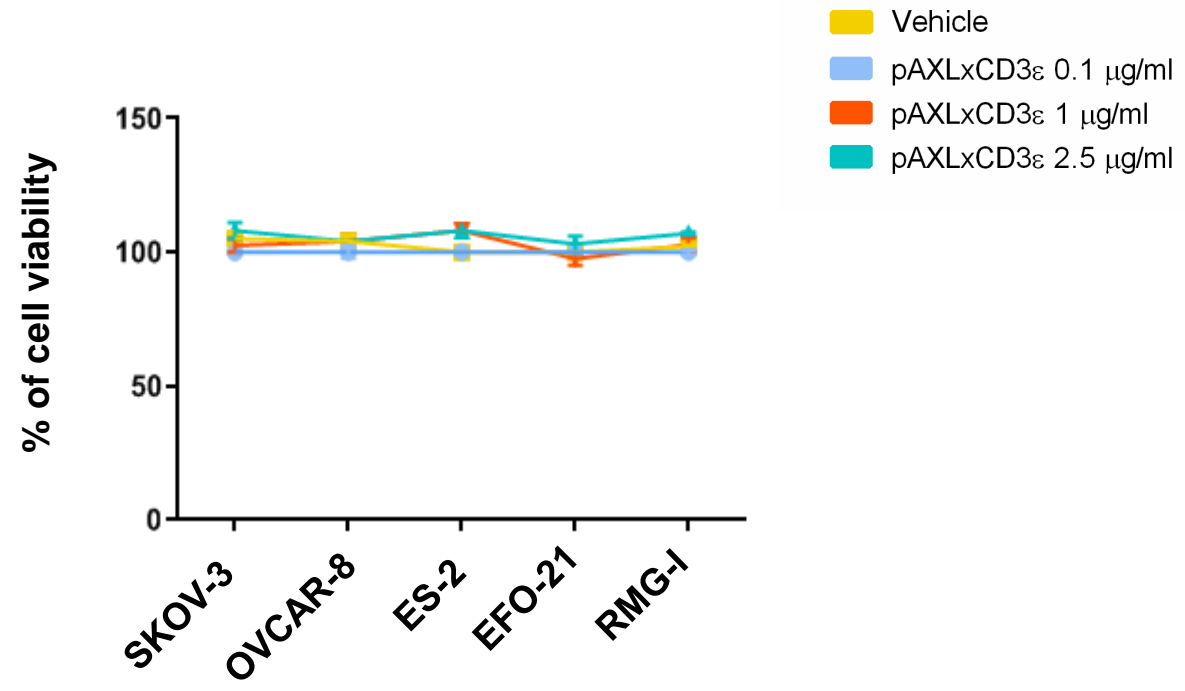

Supplement: Supplementary file 3 — Additional file 3: Figure S3. A) Percentage (%) of cell viability based on quantification of ATP present in EOC cells treated with increasing concentrations of pAXLxCD3ε (0.1 μg/ml, 1 μg/ml and 2.5 μg/ml) in the absence of effector cells. [file 12967_2023_4101_MOESM3_ESM.pdf]

**A**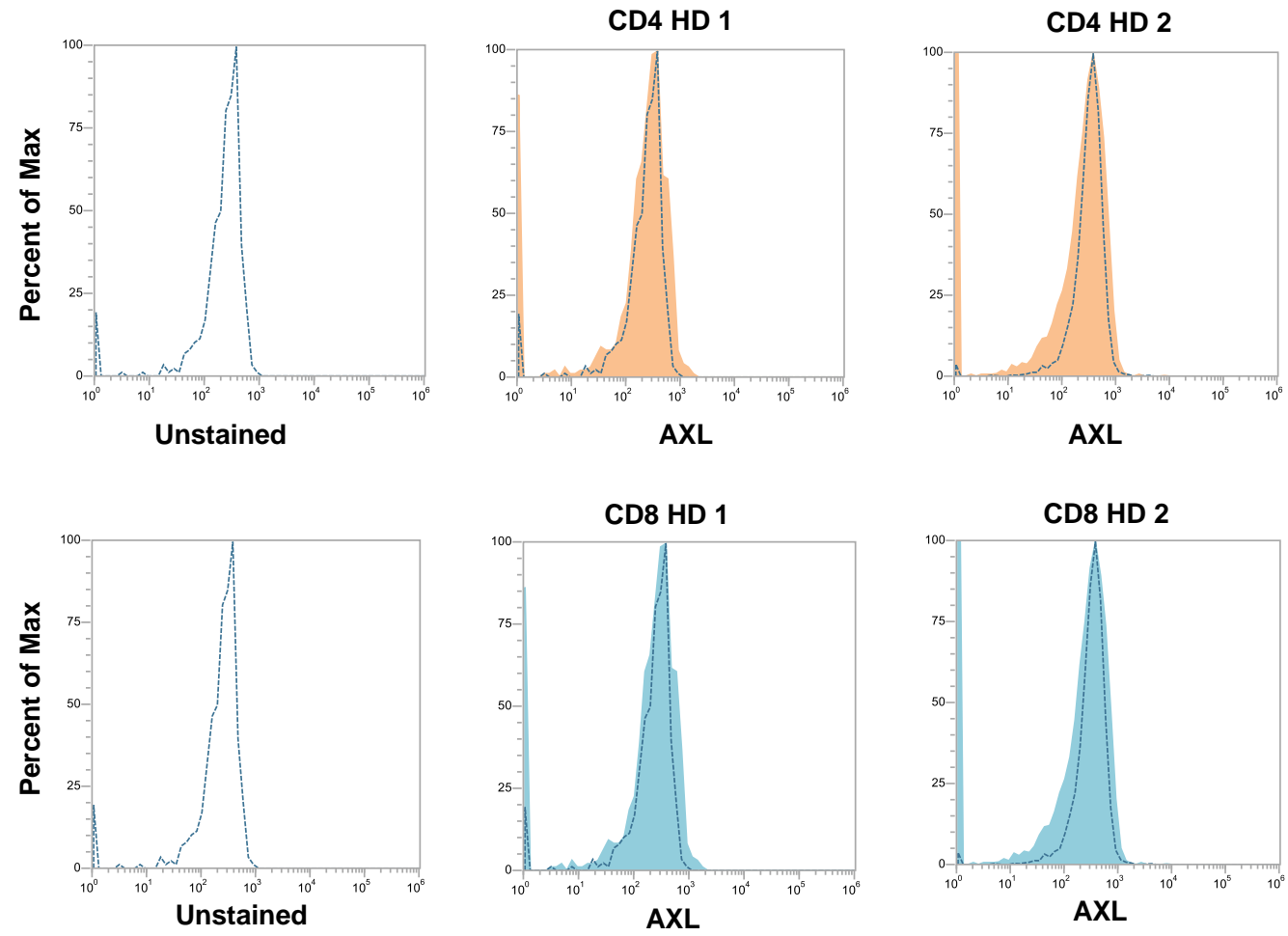**B**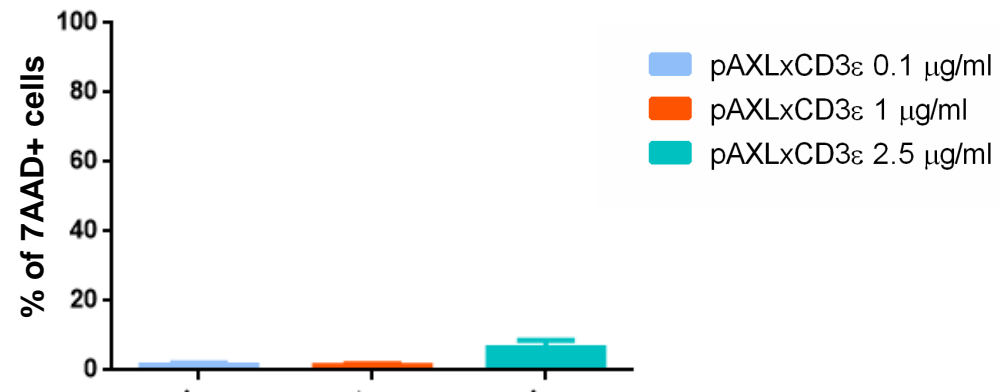

Supplement: Supplementary file 4 — Additional file 4: Figure S4. A) anti-AXL flow cytometry-based staining on CD4 and CD8 T lymphocytes from 3 different healthy donors. B) Cytotoxicity on healthy donors derived PBMCs treated for 72 hours with increasing concentration of pAXL xCD3 BTCEε (0.1 µg/ml, 1 µg/ml and 2.5 µg/ml). [file 12967_2023_4101_MOESM4_ESM.pdf]
